# Perspectives of inclusion bodies for bio-based products: curse or blessing?

**DOI:** 10.1007/s00253-018-9569-1

**Published:** 2018-12-19

**Authors:** Christoph Slouka, Julian Kopp, Oliver Spadiut, Christoph Herwig

**Affiliations:** 1 Christian Doppler Laboratory for Mechanistic and Physiological Methods for Improved Bioprocesses, TU Wien, Gumpendorfer Straße, 1a, 1060 Vienna, Austria; 2 Research Area Biochemical Engineering, Institute of Chemical, Environmental and Bioscience Engineering, TU Wien, Gumpendorfer Straße, 1a, 1060 Vienna, Austria

**Keywords:** *E. coli*, Inclusion bodies, Quality attributes, protein activity, Upstream processing, Process understanding

## Abstract

The bacterium *Escherichia coli* is a major host for recombinant protein production of non-glycosylated products. Depending on the expression strategy, the recombinant protein can be located intracellularly, which often leads to protein aggregates inside of the cytoplasm, forming so the called inclusion bodies (IBs). When compared to other protein expression strategies, inclusion body formation allows high product titers and also the possibility of expressing proteins being toxic for the host. In the past years, the comprehension of inclusion bodies being only inactive protein aggregates changed, and the new term of non-classical inclusion bodies emerged. These inclusion bodies are believed to contain a reasonable amount of active protein within their structure. However, subsequent downstream processing, such as homogenisation of cells, centrifugation or solubilisation of IBs, is prone to variable process performance and is often known to result in low extraction yields. It is hypothesised that variations in IB quality attributes are responsible for those effects and that such attributes can be controlled by upstream process conditions. In this review, we address the impact of process design (process parameters) in the upstream on defined inclusion body quality attributes. The following topics are therefore addressed: (i) an overview of the range of inclusion body applications (including emerging technologies); (ii) analytical methods to determine quality attributes; and (iii) screws in process engineering to achieve the desired quality attributes for different inclusion body–based applications. Process parameters in the upstream can be used to trigger different quality attributes including protein activity, but are not exploited to a satisfying content yet. Design by quality approaches in the upstream are already considered for a multitude of existing processes. Further intensifying this approach may pave the industrial application for new IB-based products and improves IB processing, as discussed within this review.

## Introduction

The first steps for recombinant protein expression have been made back in 1973, where Stanley Cohen and Herbert Boyer invented the possibility of in vitro DNA-cloning (Cohen et al. 1973, Baeshen et al. 2014). This opened the door for the expression of multiple diverse molecules. The first recombinant pharmaceutical product was insulin, licenced back in 1982 (Baeshen et al. 2014). Today, the main focus of the pharmaceutical market remains on the production of monoclonal antibodies, diverse hormones and growth factors, which turn out to be the majority of the pharmaceutical income (Walsh 2004, Baeshen et al. 2014, Baeshen et al. 2015).

Although complex recombinant proteins are mainly produced in mammalian cells, a good number of proteins is still expressed in *Escherichia coli* (Walsh 2014, Humer et al. 2018). Recently published papers reported that the production of biopharmaceutical proteins in *E. coli* moved up to a number as high as 40 % (Walsh 2010, Gupta and Shukla 2016). Protein production in *E. coli* gained importance as the demand in single-chain antibody fragments, which can be successfully expressed in *E. coli*, increased (Spadiut et al. 2013). *E. coli* is most likely the cheapest organism to cultivate, though its products are hard to purify and therefore take long durations and efforts when it comes to product purification (Berlec and Strukelj 2013). The genome of *E. coli* is known very well (Huang et al. 2012) and *E. coli* shows very fast replication rates, resulting in high cell densities (Murarka et al. 2007, Sahdev et al. 2007). Also, cultivations can be carried out on comparatively cheap media, coupled with the little risk of contamination compared to other cultivation hosts (DeLisa et al. 1999). In addition, scale-up can be straightforward, when compared to other organisms. Summing up, protein production using *E. coli* as a host provides a very useful alternative to mammalian cell cultivations (Baeshen et al. 2015), as e.g. yields up to 4 g/L of soluble antibody fragments have been reported already (Gupta and Shukla 2016).

Besides the classical aim to produce a high amount of protein or even toxic intermediates, recent development showed that the application range of IBs is far wider than to be expected at first glance. New approaches towards usage as biocatalysts and as nanoparticulate matter are emerging as a result of discovering protein activity in inclusion bodies (IBs). These developments make the production of active IBs and the adjustment of their physical-mechanical properties in the upstream even more important. In the last decade, the term of non-classical IBs (ncIBs)—IBs with residual protein activity—was widely used in literature (García-Fruitós 2010). However, based on many studies in the recent years, it got clear that there is no distinct borderline between classical and non-classical IBs anymore. Different approaches during strain development and especially cultivation make it possible to adapt protein activity and other quality attributes in the respective IB to a certain extent. In this review, we want to focus on the impact of upstream process parameters on different quality attributes (QAs) of IBs. Different IB producing concepts are presented in the beginning and the used analytics is compared. Following topics are therefore addressed: (i) an overview of the range of inclusion body applications; (ii) analytical methods to determine quality attributes and to sum the topic up; (iii) screws in process engineering to achieve the desired quality attributes for different inclusion body–based applications.

## Applications of inclusion body–based technologies

**Fig. 1:**
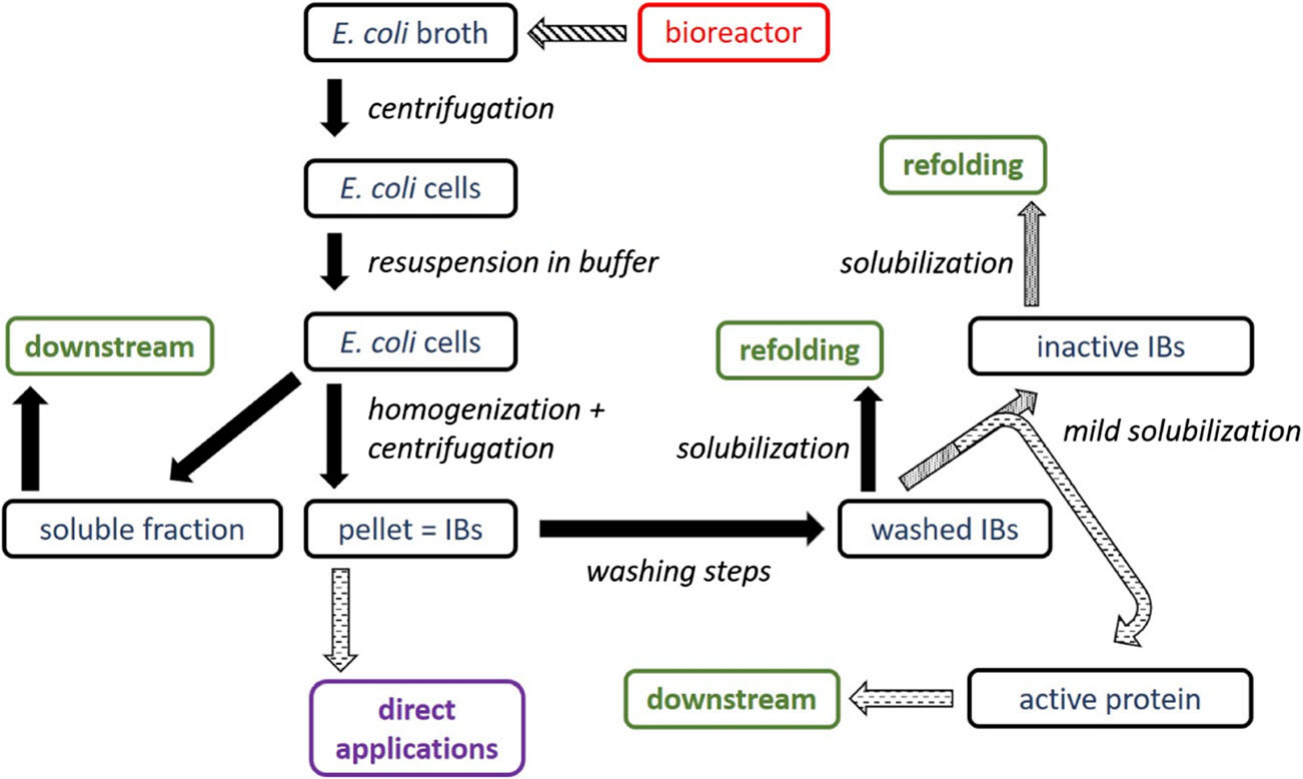
Workflow of IB production with link to different further used process steps during upstream and downstream

Inclusion bodies have originally been believed to be waste products by bacteria (García-Fruitós et al. 2012), until it was realised that they are formed as a stress reaction by the cells resulting in a supposedly biologically inactive precipitated protein (Palmer and Wingfield 2012, Ramón et al. 2014, Villaverde et al. 2015). Protein aggregation is a complex reaction, as aggregates are only formed within the same kind of proteins or highly similar proteins (Singh et al. 2015). These aggregates have to be processed to obtain correctly folded product. Figure 1 presents the general IB production and processing strategy.

### Products from IBs

Generally, approaches to express the active soluble protein in *E. coli* yield a high amount of IBs as ‘by-product’. In the last years, the notion of IBs as unwanted by-product changed fundamentally in case of high-value pharmaceutical products. *E. coli* is attracting notice as fragmented antibodies (Fabs) could be successfully expressed in the periplasm or produced in high concentration as IBs (Spadiut et al. 2013, Humer et al. 2018). IB-based processes not only enable a high production of the desired pharmaceutical ingredient, but also allow the expression of toxic proteins within the cell as no enzyme activity is given in classical IBs. Combined with the fact that IBs can be produced in that high excess (so that the amount of generated product often outweighs the downstream process (DSP) efforts), IB-based processes are believed to fundamentally boost time/space yields for recombinant protein production (García-Fruitós et al. 2012, Berlec and Strukelj 2013, Baeshen et al. 2015, Gupta and Shukla 2016). Many recent studies show that IBs consist of up to 50% correctly folded protein in contrast to the general perception of IBs as inactive structures (Jevševar et al. 2005, Peternel et al. 2008a). Already in studies in the late 1980s, IBs were already recognised to inherit residual activity when expressed at certain conditions (Worrall and Goss 1989, Tokatlidis et al. 1991). The mindset in producing IBs changed, as it was shown in a multitude of studies that IBs have a high amount of active protein being highly functional (Hrabárová et al. 2015). Enzyme tests within this study revealed that IB fractions provide high activity in classical enzyme assays when being compared to soluble (correctly folded) fractions. Trying to achieve high enzyme activity, it was proposed to create highly dense and pure IB fractions (García-Fruitós 2010). Ling et al. (Ling et al. 2015) presented approaches towards the expression of papain-like cysteine proteases. Different protocols were compared in order to achieve high amounts of active protein. However, no satisfactory process could be established yet. Kischnick et al. (2006) produced major wasp allergen antigen 5 in an IB process for an industrial-based application. Gundinger and Spadiut (2017) produced recombinant HRP in *E. coli* with optimised conditions for soluble protein (pET39b^+^ using SRP translocation pathway) and for IBs (pET21d^+^). Yield and activity of the refolded IB product were outstandingly better than the active translocated product (20 times in yield, 5 times in activity). A similar approach was performed by Jong et al. (2017) aiming for the expression of a human epidermal growth factor (hEGF) into the periplasmic space. No translocation was observed but the construct of ssTorA/hEGF (including multiple repeats of TorA) boosted IB formation in the cytoplasmic space and resulted in high yields of the desired product in the cells. Fusion tags linked to the protein of interest often favour the expression of the desired protein as IB. The N^Pro^ fusion tag (N-terminal autoprotease derived from classical swine fever virus) by Achmüller et al. (2007) not only pulls the protein to IBs, but also makes the capturing of the protein very easy. Furthermore, the fusion tag guarantees an authentic N-terminus of the protein after cleavage. Variance in temperature, induction time and inducer concentration was performed by Akbari et al. (2015) producing a single-chain antibody fragment. Optimised cultivation conditions were found at 37 °C, post-induction time of 10.45 h and 0.75 mM β-D-1 thiogalactopyranoside (IPTG) producing up to 236 mg/L of the product as IB. However, experiments were exclusively performed in shake flasks exhibiting cell densities below 2 g/L. Screening for different G protein–coupled receptors by Michalke et al. (2009) showed that fed-batch-based cultivation increased the titer of different products by at least a factor of 5 compared to classical shake flask approaches. Results obtained by Manderson et al. (2006) showed different results. The specific productivity of a recombinant vaccine against hydatidosis (Eg95) could not be increased by fed-batch-based cultures compared to shake flasks on optimised media. Interestingly, the production of Eg95 in fed-batch cultivation could be adapted by dissolved oxygen control, revealing that a dO2 of 50% is the optimum.

### Direct application of IBs

Besides pharmaceutical applications, IBs (and especially the protein activity in IBs) recently came into focus upon the establishment of bio-scaffolds for mammalian cell orientation. Not only an improved adhesion of the cells is desired but also delivering active proteins to the cells is possible (Rodríguez-Carmona and Villaverde 2010). Seras-Franzoso et al. (2013) used IBs based on human growth hormone or human chaperons for growth stimulation. These nano-active materials are supposed to release active substances in the cytoplasm and the nuclear compartments of the cells and are called nanopills. After expression and washing steps, catalytic active IBs could be directly used as catalysator for different synthesis steps often in combination with entrapment of the produced IBs. Sans et al. (2012) expressed fuculose-1-phosphate aldolase in *E. coli.* The produced IBs showed activity (in the best-case cultivation with optimised media and strain) similar to the soluble fraction. Entrapped IBs (Lentikat particles) could be successfully used as active immobilised biocatalysts. Nahálka et al. (2008) produced IBs fused to a cellulose binding domain of *Clostridium cellulovorans* for sialic acid synthesis*.* The fusion with CBM effectively promoted the aggregation of inclusion bodies in the place of soluble protein. A stable catalytic activity over 20 circles was given using the immobilised encapsulated IB. Han et al. (2017) presented that IBs could be used as active centres for metabolic engineering in *E. coli*. Heterologous enzymes for production of 1-butanol in combination with carbon binding domain interacting through a leucine zipper motive (prey-bait system) yielded in active IB for 1-butanol production even in vivo. The yield of 1-butanol production in *E. coli* could be increased by 1.5-fold using batch fermentation approaches (García-Fruitós et al. 2011, Villaverde et al. 2015).

## Analytics of IB quality attributes

Different industrial aims and applications impose variable needs of the quality of the produced IBs. The quality of a pharmaceutical product has to be very high and the effects of the upstream on the downstream are an important factor. In contrast, IBs for emerging applications need to have a high activity combined with mechanical stability during the given process. We want to address differences in the quality attributes and their measurement based on the technological application.

### Size as QA

Physiological parameters like size and morphology are of high importance for further processing steps in the downstream process (DSP) especially for the IB manufacturing in high-value production chains. Quality attributes for IBs have already been defined in several studies (García-Fruitós et al. 2012, Reichelt et al. 2017b, Wurm et al. 2017b). First approaches towards IB sizing during the induction phase were already made by Reichelt et al. (2017b) using transmission electron microscopy (TEM) in combination with nanoparticle tracking analysis (NTA) and revealed general trends of IB growth during cultivation. Wurm et al. (2017b) showed a strong correlation between induction strength (specific lactose uptake rate) and IB size and IB titer. Analysis was performed using scanning electron microscopy on a fixed harvest time point. Generally, saturation in IB size is observed around 700 nm depicting the limit of IB growth inside *E. coli*. A maximum size of 600 nm was found for the GFP model protein. Diez-Gil et al. (2010) used dynamic light scattering for IB size (and zeta-potential) measurements. Morphological analysis was performed by means of atomic force microscopy (AFM) and optical fluorescence microscopy for deposited IBs. Peternel et al. used a combination of TEM and SEM methodology for different cultivated and washed samples (Peternel et al. 2008b).

### Purity as QA

High purity is generally found at large IB size, which indicates that the surface to volume ratio is very important in receiving purer products. It is believed that major concentration impurities are a result of host cell fragments upon cell disruption. As IB formation is a very specific process for each target protein, the purity patterns for IBs might be highly different. As aggregated proteins might contain different host cell proteins, as well as RNA fragments, it is extremely important to purify these contents of the desired protein (Singh et al. 2015). Generally, SDS-PAGE and circular dichroism measurements are used for this purpose. As IBs show very bad solubility in water, polar washing procedures may help to increase purity (Fahnert et al. 2004). Addition of diverse detergents (such as Triton X-100) in low concentrations might additionally solubilise outer membrane proteins and therefore increasing purity (Clark 2001). Differentiation between impurities can be generally made using attenuated total reflectance Fourier-transform infrared spectroscopy (ATR-FTIR) measurements or even using MALDI-ToF analysis (Molloy et al. 2000, Margreiter et al. 2008). Exemplarily, Jürgen et al. (2010) performed a 2D gel electrophoresis study in combination with MALDI-ToF mass spectroscopy and showed that the quality of recombinantly produced α-glucosidase is not affected by media, cultivation mode (shake flask vs. fed-batch), sampling time, promotor system and strain background. Chip-based technologies, like Bioanalyzer measurements, result in quick and straightforward purity pattern analysis of the analysed IB (Slouka Christoph 2018). Further promising methods for analysis of purity pattern in IBs (also in combination with size determination) are coupled atomic force microscopy with infrared analysis (AFMIR) (Dazzi et al. 2012).

### Protein activity as QA

Knowledge of the amount of active protein in the IB is of high interest during the cultivation and further processing steps. In general, GFP model proteins, known to inherit protein activity, are tested using fluorescence-based methods. These could be used as integral techniques measuring the entire fluorescence of an IB sample, e.g. on a plate reader-based, or using fluorescence microscopy-based techniques for a detailed description of the IB inside the cell (García-Fruitós et al. 2007, Govers et al. 2014). García-Fruitós et al. (2007) showed that protein activity is not homogenously distributed within the structure of an IB using confocal microscopy. Peternel et al. (2009) used a multitude of different techniques for a description of the overall IB quality and activity. The purity of the soluble fraction as well as of the IB samples was measured using classical SDS-Page techniques. Folding quality was determined using circular dichroism. Peternel et al. (2008a) used similar extraction buffers for mild homogenisation with 0.2% *N*-lauroyl-sarcosine for four different products and subsequently measured the biological activity of all fractions with infrared spectroscopy (IR) in the amid I and amid II region. Jevševar et al. (2005) also used IR for determination of structural activity in their produced protein. Biological activity could be found in all IB samples but differed greatly depending on the protein. However, very low solubility of the active part of these proteins was observed using the described mild solubilisation method, indicating that solubilisation and protein activity in IBs was highly dependent on the product. Enzymatic conversion activity can be used to show the activity of the IB as biocatalysts. Photometric analysis of IB activity was used by Sans et al. (2012) measuring the dihydroxyacetone phosphate salt using an UV/VIS photometer. Other metabolites during the reaction were determined using online HPLC methods. Nahálka et al. (2008) measured conversion kinetics by means of flow calorimeter techniques. After calibration using HPLC, the conversion efficiency could be directly detected via a voltage signal of the thermistor.

## Impact of the upstream process on IB QAs

A broad range of proteins is produced as IB for high concentrations of the desired product or due to the use of IBs as biomaterials or biocatalysts (Villaverde et al. 2015, Rinas et al. 2017). Generally, stress due to strong overexpression of foreign DNA (Fahnert et al. 2004) in combination with a slow folding machinery is believed to lead to protein aggregation during cultivation (Thomas and Baneyx 1996). Stress as high temperatures, pH shifts or high feeding rates also favour IB formation (Fahnert et al. 2004). These factors tend to result in higher yields of product (Gupta and Shukla 2016), which of course is advantageously combined with the possibility of expressing toxic proteins (Berlec and Strukelj 2013). We want to focus on different screws for altering QAs of IBs starting with the choice of the strain and the induction mechanism, classical process parameters like T and pH and finally physiological feeding control. An overview of the impacts of upstream process parameters on IB QAs reported in the literature to date is given in Table 1. Classical process parameters, like temperature and pH, have a severe influence on the expression of IBs and/or soluble protein. However, many studies are only based on shake flasks experiments or only batch approaches, being far away from realistic biomass concentrations in industrial applications. Table 2 differentiates effects in IB QAs based on the process design (shake flask vs. bioreactor cultivation). The uncontrolled matter of shake flasks (no direct pH, dO_2_ control, no fed-batch approaches) often leads to differences in the IB expression, but usually has almost no influence on product QAs. In most basic science studies, and especially for emerging technologies, shake flasks represent an easy straightforward cultivation technique. However, based on regulatory instances and QbD criteria, bioreactor cultivations (generally stirred tank reactors) are today’s state of the art for microbial cultivation. Therefore, special focus is laid on the effects on IB QAs during bioreactor-based upstream.

**Table 1:** Differences in QA and performance indicators for different IB-based products. QAs can be influenced by process parameters in the USP

Product	Quality attributes (QA)	Key performance indicator (KPI)	Screws in critical process parameters (CPP)
IBs without protein activity	• Size (Peternel et al. 2008b, Díez-Gil et al. 2010, Rinas et al. 2017, Slouka Christoph 2018) • Purity (Kischnick et al. 2006, Slouka Christoph 2018)	• Time-space yield/titre • DSP performance	• Classical (pH, T, pO_2_) (Castellanos-Mendoza et al. 2014, Slouka Christoph 2018) • Physiological (*q*_s,C_) (Slouka Christoph 2018) • Cell viability (Reichelt et al. 2016, Slouka Christoph 2018) • Inducer (e.g. IPTG vs lactose) (Neubauer and Hofmann 1994, Akbari et al. 2015, Wurm et al. 2016, Wurm et al. 2017b) • C-source (Kopp et al. 2017) • Induction time (Rinas et al. 2017) • Dissolved oxygen (Manderson et al. 2006)
IBs with protein activity	• Size • Purity • Activity (García-Fruitós et al. 2007, Govers et al. 2014) (Peternel et al. 2009)	• Recovery of mild solubilisation performance • DSP performance	• Classical (pH, T) (Castellanos-Mendoza et al. 2014) (Jevševar et al. 2005) (Peternel et al. 2009) • Fusion tags (Wang et al. 2015) • Induction time
IBs as nanoparticulate matter	• Size • Cell penetration (Seras-Franzoso et al. 2013) • Drug delivery (Rodríguez-Carmona and Villaverde 2010)	• Mammalian cell growth	
IBs as biocatalysts	• Catalytic activity (Nahálka et al. 2008, Sans et al. 2012) • Recirculation number (Nahálka et al. 2008) • Purity	• Turnover number	• Fusion tags (Nahálka et al. 2008)
IBs for multienzyme cascades	• Catalytic activity (Han et al. 2017)	• Metabolon activity

**Table 2:** Differences between shake flask expression and fermenter cultivation for different QAs and KPIs

Effect on IB QA	Shake flask	Fermenter	Citation
IB size of sphingomyelinase-D	Higher than 500 nm	Lower than 500 nm	Castellanos-Mendoza et al. 2014
Specific productivity of sphingomyelinase-D after 24 h	0.32 ± 0.04 *g*_prot_/*g*_X_	0.48 ± 0.03 g_prot_/g_X_	Castellanos-Mendoza et al. 2014
Effects on composition of α-glucosidase	Identical	Identical	Jürgen et al. 2010
Titre of G protein–coupled receptors after capture	–	Five- to tenfold increase	Michalke et al. 2009
Spec. productivity of Eg95	Slightly better	–	Manderson et al. 2006
Secondary structure of β-lactamase	Identical	Identical	Margreiter et al. 2008

### Impact of induction system on IB QAs

There are a multitude of promotor systems, which are regularly used in *E. coli*. The most important for therapeutic protein production are lac, lac-trc or T7 promotors, which are commonly induced by isopropyl β-D-1 thiogalactopyranoside (IPTG) (Neubauer and Hofmann 1994, Wurm et al. 2016). However, induction with IPTG stresses the cells (Neubauer and Hofmann 1994, Viitanen et al. 2003, Marbach and Bettenbrock 2012, Dvorak et al. 2015). Alternative induction mechanisms are temperature shifts (*λ*-phage), phosphate depletion (phoA) (Huang et al. 2012) and induction with L-rhamnose (rhaT). In contrast to most of the other promotor systems, rhaT is tightly regulated and renders turnability of recombinant protein production possible (Giacalone et al. 2006). This is most likely due to a positive-coupled feedback system, when compared to the commonly used T7-expression system (Wegerer et al. 2008) and may be a powerful tool to modify IB QAs based on the induction strength. Similar to the rham-BAD-system, induction is performed with arabinose using an ara-BAD-System, hence suffering from the same drawbacks as the rhamnose induction system, (Khlebnikov et al. 2002) being the usually high cost of l-rhamnose and l-arabinose. Comparison of protein expression of eGFP based on induction with IPTG and lactose has been performed by Wurm et al. (2017b). Induction with lactose (using a pET vector system) gives the possibility to adapt the QA size and purity for the given inclusion body based on the inducer uptake rate. Akbari et al. (2015) studied the effects of the inducer concentration using IPTG in a pTZ57R/T cloning vector. Changing the inducer concentration showed a significant impact on the protein production analysed in combination with temperature and induction time. Kischnick et al. (2006) observed that a component of papain digested soy peptone mimics the effects of IPTG and may therefore be used as a cheap alternative in complex media systems.

Besides the classical parameters, physiological control of the cultivation process is important for optimisation of the KPIs and QAs. Physiological feeding strategies are based on the specific substrate uptake rate (*q*_s,C_) of the respective C-source and can be exclusively performed in the controlled environment of a bioreactor. Cell stress and reduction of cell viability during the induction lead to a reduced uptake of C-source and therefore to a possible overfeeding during induction (Reichelt et al. 2016, Slouka Christoph 2018). Adapting of *q*_s,C_ based on the physiological state of the cell is essential for preventing cell death and IB degradation during cultivation. Furthermore, IPTG as inducer often imposes high cell stress during induction. Application of lactose for T7-based systems as inducer is a method to prevent this high imposed stress, since lactose can be metabolised during induction. Further benefits of lactose are the possibility to adapt induction strength in a mixed feed approach varying *q*_s,C_ of the C-source and lactose, respectively. Wurm et al. (2017a) tested different recombinant proteins for concomitant uptake of glucose and lactose. Results show that IBs are strongly dependent on the amount of the specific lactose uptake rate in the mixed feed and on the specific uptake rate of the primary carbon source. Furthermore, these cultivation techniques help to overcome the problem of cell stress caused by harsh induction using IPTG and render the possibility for tuning between soluble and IB production. Mixed feed could be applied for a multitude of products including antibody fragments (Fabs) (Wurm et al. 2017a) and can be used to increase the production of soluble protein compared to the IB fraction (Wurm et al. 2016), also valid for a GFP model protein, expressing IBs as well as soluble protein (Wurm et al. 2017b). Also, effects on the activity of the produced GFP were visible within this study. Changing the primary carbon source from glucose to glycerol in mixed feed systems with lactose was performed by Kopp et al. (2017), though no major differences between glucose and glycerol uptake regarding the specific inducer uptake rates could be detected. The specific IB productivity was highly increased using glycerol as the primary carbon source, which makes the cheap carbon source an ideal alternative for glucose (Martínez-Gómez et al. 2012).

### IB size affected by process parameters

The first important QA to be discussed is IB bead size. The high correlation between IB titer and purity is generally known. Size is believed to be highly affected by the expressed protein, e.g. amino acid sequence, protein tags and hydrophobicity. In several studies, IB growth is believed to be limited in a certain size, which is often about 700 to 800 nm in diameter.

Effects on IB size through alterations in the culture pH was investigated by Castellanos-Mendoza et al. (2014) producing recombinant sphingomyelinase-D. The cells were cultivated in a shake flask and batch approaches. Differences in size were detectable during the 24-h runs with usually higher size present in the uncontrolled shake flasks experiments. However, controlled pH conditions favoured higher product yield. Performed fed-batch-based cultivations using an industrial protein fused to N^Pro^ exclusively expressing IBs (Slouka Christoph 2018) showed high impact on IB size based on classical process parameters tested in a design of experiments (DoE) approach. A low pH of 6.7 and a low temperature of 31.5 °C during the process favour IB productivity in an exclusively IB-producing strain. Besides specific titer, also QAs of size (analysed with SEM) and purity were analysed and optimised within this study. IB sizing during the induction phase was also made by Reichelt et al. (2017a, b) altering the induction time and analysing the size dependence with different methods. They showed that higher titers and longer induction timers increase the overall IB size. Expression of GFP as IB by Wurm et al. (2017b) showed a strong correlation between induction strength (specific lactose uptake rate) and IB size. A maximum size of 600 nm was found for the GFP model protein, which is strongly induction level–dependent. Others report IB sizes between 502 nm for DnaK, IBs and 580 nm for ClpA-IBs (Díez-Gil et al. 2010) and approximately 600 nm for G-CSF IBs (Peternel et al. 2008b). These studies show that there seems to be a maximum IB size in *E. coli* cells, which is approximately 700 nm. As IB formation is a highly a time-dependent process, IB size can be triggered via harvest time point alteration (Rinas et al. 2017).

### IB Purity affected by process parameters

Generally, purity as QA is only accessible after cell disruption of the host and subsequent measurement of the IB with different proposed methods. Cell disruption tends to be a cause for a high amount of impurities in recombinant protein, especially in IBs, since hydrophobic substances, especially membrane proteins, of the host cell tend to accumulate at the surface of IBs. The library of outer membrane proteins in *E. coli* including mass, isoelectric point and hydrophobicity, was measured with 2D gel electrophoresis and subsequent MALDI-ToF SIMS (Molloy et al. 2000).

Jürgen et al. (2010) showed that different intercellular proteins are situated within the structure of IBs, making them an inhomogeneous structure. Several impurities were found within this study: the IB-associated proteins IbpA and IbpB, the kanamycin resistance protein was found to be associated with the α-glucosidase-IBs. Finally, besides the chaperones DnaK and GroEL, the outer membrane protease OmpT was found in the aggregated protein fraction. Besides the accumulation of impurities within the aggregation of IBs inside the cell, homogenisation of the host imposes a further source for impurities on top of IBs as already presented by Rinas et al. in the 1990s (Rinas and Bailey 1992). Some process parameters are found to influence purity pattern in IBs. Low T is favourable for IBs of high purity tested in a DoE approach between 30 and 40 °C induction temperature by Slouka et al. (2018) for two tested proteins. Induction temperature had also a high influence on disulphide bonding content in IBs of native recombinant β-lactamase presented by Margreiter et al. (2008). Despite different cultivation techniques, a purity of about 95% was found within this study. Limited oxygen growth conditions yielded increased productivity and purity for an IB for wasp allergen 5–based process by Kischnik et al. (2006).

### IB activity affected by process parameters

Different process parameters were recently identified to increase the expression of active structures within produced IBs. Castellanos-Mendoza et al. (2014) showed that changes in the culture pH changed the IB characteristics of sphingomyelinase-D between the classical and active form. Uncontrolled pH resulted in a higher amount of active IBs analysed by means of solubilisation and enzyme kinetic tests. To increase the amount of biological active inclusion bodies, Wang et al. (2015) tested different hydrophobic self-assembling polypeptides fused to the C-terminus of two model proteins—*Bacillus subtilis* lipase A (LipA) and *Aspergillus fumigatus* amadoriase II (AMA). Results indicated that these protein tags increase the amount of the insoluble fraction drastically, with remaining activity. Jevševar et al. (2005) increased the fraction of active IB in human granulocyte colony-stimulating factor (hG-CSF) by decreasing temperature to 25 °C during induction. This yielded in an increased titer (higher than 2-fold) and an increase of biological activity up to 30%. Peternel et al. (2009) produced GFP as a model protein and altered induction strategy and induction temperature. Higher titre could be found under conditions of delayed induction. As already shown in different studies, lower temperature also increased the amount of active protein within IBs.

Higher activity in IBs may fundamentally increase the overall DSP yield, when extracted prior to refolding (compare to Fig. 1). IBs for direct applications or catalytic purposes are up to now only produced in shake flask experiments or in small-scale batch cultivations using only a few controls. Process engineering approaches may be highly beneficial in the production of distinct bead sizes for scaffolds and in boosting the activity of catalytic active IB materials.

## Understanding and design criteria

**Fig. 2:**
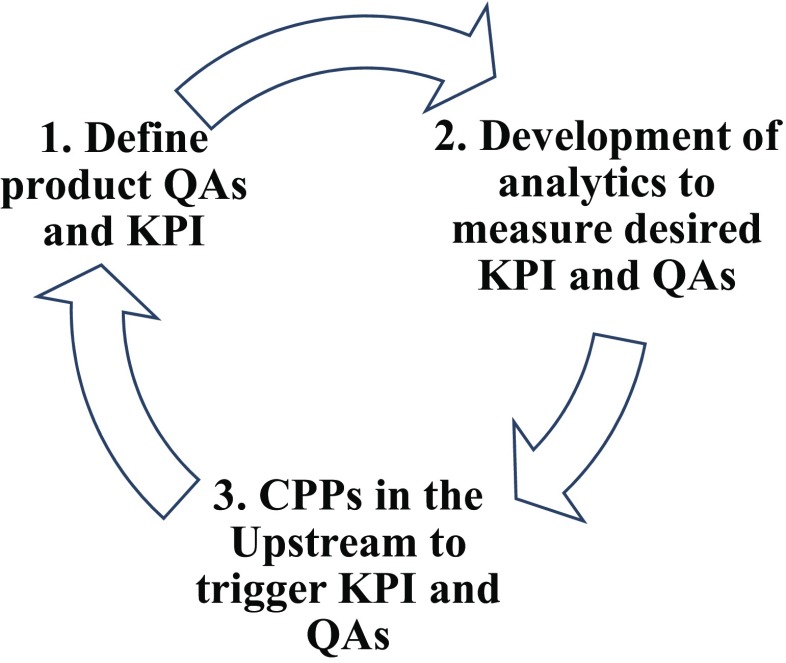
Product development chain for (Singh et al.) products and its iterative optimisation process

The IB-based applicational range widened remarkably during the last decade, mainly based on the findings of the biological activity of IBs. Product design and expression are only one important part in the product development chain. Figure 2 presents the general approaches for optimisation of IB-based production processes based on the presented chapters in this review. After conceptualisation of the product and the expression system, the development of respective analytics for measurement of the critical QAs is of utmost importance. For pharmaceutically applied products, titer and purity measurements during the downstream (chromatographic steps) are already implemented. However, without direct analysis—especially in a time-dependent manner—in the upstream, control during the process is challenging. Therefore, several analytical methods presented in the literature were summarised in this review and represent the most important pillar for process understanding and control.

While definitions of QAs and detailed analysis on the downstream were already given in recent reviews, e.g. (Humer et al. 2018), we focus on the effects of the upstream on IB QAs. Important QAs for different IB-based applications are summarised in Table 1. QAs, like size and purity, and KPIs, like titre, can be already well measured in the upstream process based on time-dependent sampling. Enzyme/catalytic activity measurements of the product are also extensively researched. Model proteins like GFP enable us to get inside on effects of critical process parameters (CPP) on IB protein activity and its modification during the upstream. This knowledge enables to increase the number of direct applications of IBs for enzymatic reactions in vitro and is also believed to increase downstream recovery of the product. However, in several applications, process engineering methods are not applied to a satisfying content, also to be seen in empty spaces in Table 1. Different screws in process parameters for adapting the most important QAs are already identified, but are primarily based on pharmaceutical products. Despite the knowledge of important QAs for new applications, upstream technological approaches are not engineered sufficiently in order to control KPIs and QAs. One reason may be that diverse products are in the very early stage of development for several applications. Optimisation of the process is therefore not under investigation yet as the efforts are too high. In case of industrial need for these IB-based products, based on competitive prices and quality, an emerging need for upstream screws will be given in the near future. A second reason may be investigations of IB properties intending a rather basic science claim. Summing up, bio-engineering is a powerful tool not only to broaden the application range of IB-based products in the industry, but also to improve product quality and quantity. This further process understanding for a high variety of products with different applications may also pave the way for new benchmarks in the industry, e.g. continuous manufacturing in microbial processes.

## Conclusions

IB processes experienced a new renaissance in recent years, as the range of application of IBs increased considerably. Classical process parameters, like temperature, pH and physiological feeding, in combination with different induction mechanism, are powerful tools to trigger the properties of IBs in order to fit the desired quality. IBs are still widely exploited for the production of pharmaceutical processes for high product titer and expression of toxic proteins, where no posttranslational modifications are required, e.g. Fabs.

The differentiation between classical and non-classical IBs is not a distinct border anymore. Different process parameters in the upstream do highly affect the amount of active protein within the IB. Process understanding during upstream processing in this respect is still highly underestimated. Different screws are already identified for individual products to enhance or repress activity in the IB product. Desired QAs for different applications are already triggered by process technology–based tools. While the analytical toolbox for determination is already developed to a high extent and applicable, quality by design criteria are not yet applied for a multitude of products as no need for high production is given yet. Furthermore, we believe that, if further basic research is done for a number of products, distinct process understanding during production will help to improve product quality (and quality attributes) for these IB-based products in the future.
